# Recovery of infrastructure networks after localised attacks

**DOI:** 10.1038/srep24522

**Published:** 2016-04-14

**Authors:** Fuyu Hu, Chi Ho Yeung, Saini Yang, Weiping Wang, An Zeng

**Affiliations:** 1 The State Key Laboratory of Earth Surface Processes and Resource Ecology, Beijing Normal University, Beijing 100875, P. R. China; 2 Academy of Disaster Reduction and Emergency Management, Beijing Normal University, Beijing 100875, P. R. China; 3 Department of Science and Environmental Studies, The Hong Kong Institute of Education, Taipo, Hong Kong; 4 School of Systems Science, Beijing Normal University, Beijing 100875, P. R. China

## Abstract

The stability of infrastructure network is always a critical issue studied by researchers in different fields. A lot of works have been devoted to reveal the robustness of the infrastructure networks against random and malicious attacks. However, real attack scenarios such as earthquakes and typhoons are instead localised attacks which are investigated only recently. Unlike previous studies, we examine in this paper the resilience of infrastructure networks by focusing on the recovery process from localised attacks. We introduce various preferential repair strategies and found that they facilitate and improve network recovery compared to that of random repairs, especially when population size is uneven at different locations. Moreover, our strategic repair methods show similar effectiveness as the greedy repair. The validations are conducted on simulated networks, and on real networks with real disasters. Our method is meaningful in practice as it can largely enhance network resilience and contribute to network risk reduction.

In the past decade, many studies have contributed to the interpretation of topological structures^1^,^2^,^3^, dynamical processes^4^,^5^,^6^ and controllability^7^,^8^,^9^ of complex networks. Among them, a large proportion of research explained the robustness or vulnerability of networks by percolation theory and dynamic simulation^10^,^11^, as well as critical component identification^12^,^13^. Recently, there were concerns focusing on the study of resilience of infrastructure networks against attacks or failures, which is significantly benefited from the rapid development of complex network research^14^,^15^,^16^,^17^. Substantial research focused on the random attack and malicious attack^18^,^19^,^20^,^21^,^22^, and a more realistic attack, namely the localised attack, only starts to draw attention recently. Localised attacks are geographically attacks induced by natural disasters (e.g. tropical cyclone, earthquake, landslide and so on) or mass attacks (burst of atom bomb or hazardous chemicals). The main difference between localised attacks and random attacks or malicious attacks is that the localized attacks always cause aggregated destruction of adjacent components limited to a specific area, while the random attacks or malicious attacks are global attacks and the failed components are distributed throughout the whole system. So far, localised attacks are studied on monopartite network^23^ and spatially embedded interdependent networks^24^ with the percolation theory.

The recovery and repairability of complex networks have attracted more and more attention lately. Recovery mechanism exists in nature, which includes the spontaneous recovery and deliberate repairs. Spontaneous recovery is defined as the process that damaged components of the network spontaneously become active again in a period of time after failure^25^. For example, a river bed dries up in dry seasons and the river losses its transport capacity, but it regains its water level and function in wet seasons. On the other hand, deliberate repairs refer to the procedures that the significant damaged parts in the network are manually restored when the system cannot regain its function automatically or fail to be repaired in a reasonable time. For instance, after a severe earthquake, infrastructure systems such as power-grid and transportation networks may be damaged and cannot be recovered without external efforts. In some systems these two types of recovery can co-exist, e.g., heavy snow can cause traffic congestion, which may let-up by gradual spontaneous recovery, but the duration is usually long. Snow plowing is needed as a way of deliberate repair to shorten the recovery process. Manual recovery approach for systems with high cost-benefit ratio can largely enhance the system resilience and contribute to emergency management and system risk reduction.

Robustness and repairability are two important resilience criteria^14^. Robustness is closely related to network redundancy^26^,^27^,^28^. Some researchers enhance the resilience of network through increasing the inherent redundancy by using self-healing algorithms^29^. For localised attacks, merely strengthening network robustness against attacks may not make the network resilient to some specific attacks. For instance, localised attacks always simultaneously disrupt multiple adjacent components, including both nodes and edges. In this case, the redundancy of network cannot effectively resist or absorb the disturbance since the whole local region is disrupted, and deliberate repairs play an important role to restore the system functionality. In transportation networks (especially road networks), methods to recover from these edge attacks are crucial since transportation to the disrupted region is totally distorted, and such methods will also be relevant to other systems such as power-grid and pipeline networks. To the best of our knowledge, so far there is no research on the deliberate recovery of complex networks after localised edge attacks.

The aim of this paper is to identify the optimal repair strategy on geographical networks after localised attacks. First, we will illustrate the deliberate recovery process of two-dimensional weighted square lattice under localised attacks. Secondly, four different repair strategies are devised and tested under localised, random and malicious attacks. Finally, we validate the repair strategies on a real-world road network in Hainan province in China under two real historical damage profiles caused by an earthquake and a tropical cyclone.

## Results

### Problem Statement

Specifically, we focus on the road networks and model them with two-dimensional square lattices with side length *l*, which is the simplest geographical network with *N* = *l*^2^ nodes and *E* = 2*l*(*l* − 1) edges. The network is denoted by an adjacency matrix *A* where *A*_*ij*_ = 1 if there is an edge connecting vertexes *i* and *j*, and *A*_*ij*_ = 0 otherwise. Nodes in the network correspond to regions or towns, with a population size drawn randomly from a power-law distribution. Edges in the network correspond to roads, and a fraction of them are disrupted under attacks. We call this ratio the *edge damage percentage*.

**Figure 1:**
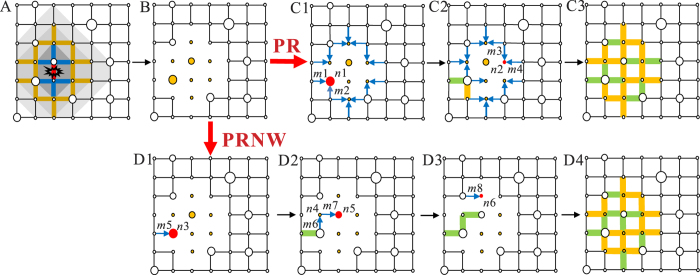
The illustration of various strategic repair processes after localised attack (LA) on two-dimensional square lattice with heterogeneously populated nodes. The attack center is randomly selected. (**A**) The schematic localised attack. The attack intensity will decline with distance from the attack center. An edge is disrupted only if the attack intensity is larger than a threshold. The distance between two edges is defined as the vertical distance from the mid-point of one edge to other edge. The darkest gray area suffers the largest attack intensity, and the lightest gray area suffers the smallest attack intensity which is lower than the physical disruption threshold of edge. Only the edges coloured red, blue and yellow fail. In this case, a group of geographically localised edges fail and are removed from the network. (**B**) The remaining functional edges after localised attack, and the yellow nodes are isolated. (**C1**–**C3**) The operations of PR. In (**C1**) the blue edges with arrowhead are the damaged edges adjacent to the functional components of the network. The red node *n*_1_ is the node adjacent to the network with the largest population, and either edge *m*_1_ or *m*_2_ will be repaired first randomly. In this case, *m*_1_ is selected to be restored first and coloured green. After all the isolated nodes are connected at last, *m*_2_ will be repaired coloured yellow. At the next step, the node *n*_2_ in (**C2**) is the node adjacent to the functional network and with the largest population, and either edges *m*_3_ or *m*_4_ will be repaired randomly. The process will be iterated until all the isolated nodes are connected to the functional network, as shown in (**C3**). At last, the yellow edges will be repaired randomly one by one until all are repaired. (**D1**–**D4**) The operation of PRNW. In (**D1**) the red node *n*_3_ has the largest population among all the isolated nodes, and edge *m*_5_ connects *n*_3_ to the network. The edge *m*_5_ will be repaired first and coloured green. In the next step, *n*_5_ is the most populated node; the edges *m*_6_ and *m*_7_ which connect *n*5 to the functional network, will be repaired one after the other. The procedures are iterated until all the isolated nodes are connected to the network, as shown in (**D4**). At last, the yellow edges will be repaired randomly one by one until the all edges are repaired.

There are several different attack scenarios. Firstly, localised attacks (LA) model natural hazards which occur in specific areas. It is a group of failed edges concentrated in a geographical domain, resulting in adjacent isolated nodes, as illustrated in Fig. 1(A,B). Secondly, malicious attacks (MA) model the case where terror attacks disrupt the most important parts in a network to damage its functionality as much as possible. In MA, the edges with the largest betweenness centrality (updated dynamically) are removed in order, leading to the emergence of several separated sub-networks, as illustrated in Fig. S1(a,b). Thirdly, random attacks (RA) model random failures. In RA, edges are damaged in random (see the illustration in Fig. S2(a,b)). Reference^30^ has pointed out that malicious attacks are more destructive than random attacks. In this paper, we aim to address the question: which recovery methods should be adopted to recover from network disruptions, especially for localised attacks?

### Network Functionality

To quantify the effectiveness of different recovery approaches, we first define *network functionality* and measure the speed and the extent various approaches recover. We introduce the *weighted inverse distance*, which is an index used to measure the efficiency of the whole network, formulated as


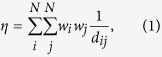


where *η* denotes weighted inverse distance; *w*_*i*_ and *w*_*j*_ respectively denote the weight or population of nodes *i* and *j*. The shortest distance between nodes *i* and *j* is denoted as *d*_*ij*_, and it is infinite if nodes *i* and *j* are not connected. We then normalize *η* by


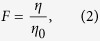


where *F* denotes the network functionality; *η*_0_ denotes the weighted inverse distance of the network before the attack. In other words, *F* = 1 if the network is intact, and *F* = 0 if the network is totally collapsed.

### Recovery Approaches

To recover the network from damages, the most straightforward method is *random recovery* (RR), where the damaged edges are repaired randomly. A more effective method is to repair the damaged links in order such that the network regains the largest network functionality in each time step. This method is called *greedy recovery* (GR). However, by doing this there is no guarantee that this order of recovered links leads to the fastest recovery when all the possible sequence of recovered links are combined. For instance, it may happen that some links which lead to sub-optimal recovery have to be repaired first, in order to achieve the fastest recovery in later steps. In other words, the method could be trapped in a local optimum since we do no search exhaustively in the space of the order of repaired links. GR has a high computational complexity and is very difficult to be applied to large scale networks. Thus, local optimal strategies become preferable. Figure 1(C,D) respectively depicts the processes of two local strategies under LA. The first method is called *preferential recovery based on nodal weight* (PRNW), whose main idea is to preferentially repair the edges which connect the isolated nodes with the largest population to the functional component of the network. The other method is called *periphery recovery* (PR). As LA always results in a group of adjacent isolated nodes, the isolated node with the largest population at the boundary has the priority to be repaired. The recovery procedures by PRNW and PR under MA and RA are respectively illustrated in Figs S1 and S2. Both the greedy recovery and two local approaches are deliberate strategic recovery methods, which means that links are restored in a specific order to speed up the recovery of the damaged networks. The mathematical representation of above recovery approaches has been shown in the Methods section.

### Recovery Metrics

In order to measure and compare the effectiveness of the various recovery approaches, we define the following quantitative measures: edge recovery percentage, recovery level and recovery efficiency.

Edge recovery percentage (*ζ*) is the ratio of damaged edges restored after attack, as Equation (3).


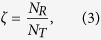


where *N*_*R*_ denotes the number of restored edges; and *N*_*T*_ denotes the total number of damaged edges.

Recovery level (*RL*) is the degree to which the network regains its functionality after some damaged edges are repaired, given by


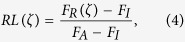


where *F*_*I*_ denotes the network functionality of the damaged network after attack; *F*_*A*_ denotes the intact network functionality before attack; and *F*_*R*_(*ζ*) denotes the network functionality after a percentage *ζ* of damaged edges are repaired. After all, *RL*(*ζ*) denotes the recovery level after a percentage damaged *ζ* of edges are repaired. Therefore, the value of *RL* is between 0 and 1. If the damaged network has not began to be restored yet, *RL* = 0; If the network has been totally repaired well, *RL* = 1;

Recovery efficiency (*RE*) measures the effectiveness of the recovery approaches, formulated by the integral of the recovery level *RL*(*ζ*) in Equation (4), given by


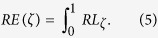


We denote *RE*_*PRNW*_, *RE*_*PR*_, *RE*_*RR*_ as the recovery efficiency of PRNW, PR and RR respectively.

### Numerical Simulations

**Figure 2:**
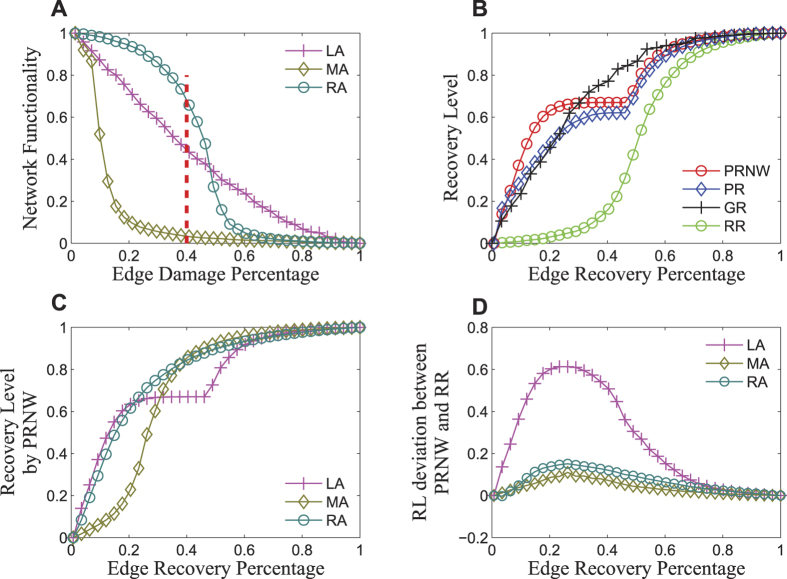
The change in network functionality and recovery level after the attack and recovery in two-dimensional square lattice with heterogeneous population on individual nodes. (**A**) The degradation of network functionality as a function of the percentage of damaged edges under LA, MA and RA. The red dotted line in the figure corresponds to the percentage of damaged edges, i.e. 0.4, from which restoration processes start. (**B**) The recovery level as a function of the percentage of repaired nodes of PRNW, PR, GR and RR after LA. (**C**) The recovery level as a function of the percentage of repaired nodes of PRNW after LA, MA and RA. (**D**) The difference of the recovery level between PRNW and RR after the three kinds of attack.

The numerical simulations are conducted on two-dimensional square lattices with *l* = 50 and *N* = 2500 and heterogeneous population on nodes. Figure 2(A) shows the decrease of network functionality with the percentage of damaged edges. Under MA, the network loses most of its functionality in the early stage because the removed edges are the most critical ones. The decreasing speed of the network functionality induced by RA is small when the damage percentage is less than 50% but becomes significantly larger afterwards. This is due to the emergence of isolated components in the network. The decrease of network functionality under LA seems to be linear and the decreasing rate is stable without large variation at any edge damage percentage.

Figure 2(B) shows the network recovery level under LA after removing 40% of links, i.e., the initial percentage of damaged edge is 0.4. As we can see, the various strategic recovery approaches are significantly more effective than random recovery to recover network functionality. GR aims at repairing the best link at each step, in the sense that the increase of network functionality is maximum at each step. The recovery level quickly reaches a very high value around 0.7. In RR, the recovery level increases first very slowly but becomes fast when the edge recovery percentage is larger than 0.5. There are *l*^2^ nodes and 2*l*(*l* − 1) edges homogeneously distributed in the lattice network. Under LA, after damaging *P*% of the edges, there will be roughly *P*/2% isolated nodes concentrated in a specific domain. Connecting all the isolated nodes needs at least *l*^2^ *P*/2% links, which is very close to 50% of the damaged edges. It means that the network becomes connected again when edge recovery percentage reaches at least 50%. Therefore, the recovery level by RR stays very low when the recovery percentage is small but increases substantially after the recovery percentage is higher than 0.5.

In contrast to GR and RR, the recovery level of PRNW and PR increases non-linearly with recovery percentage. PRNW and PR have three points with abrupt changes. As for PRNW, the three points respectively locate at edge recovery percentages around 0.2, 0.5 and 0.7, which divides the recovery process into four stages. In the first stage, the damaged edges connected to the most populated nodes are first repaired; the recovery level increases quickly. In the second stage, the damaged edges linking the less populated but isolated nodes are repaired and the network becomes connected at an edge recovery percentage of 0.5. These links between the connected component and the isolated nodes with small weight contribute less to the functionality of the network, and hence the increase of recovery level is very small at this stage. However, the connectivity of network is restored at this stage, which means that network recovers its basic functionality again. In the third stage, the increase of recovery level becomes faster because the edges restored in the second stage effectively reduce the shortest distances. At the final stage, the recovery level reaches a high value, and only the remaining edges between less populated nodes are repaired. These links are in many cases redundant.

For PR, the three points of abrupt changes are located at edge recovery percentages around 0.3, 0.5 and 0.7. There also exist four stages and the latter three stages are very similar to those of PRNW. As for the first stage, repairing is prioritised for periphery edges linking an isolated node with large population. The recovery level also increases quickly. Since population size follows a power-law distribution, the number of nodes with large weight is far less than those with small weight. In the initial steps, when edge recovery percentage is smaller than 0.1, the recovery level of PR is larger than that of PRNW. The reason is that in PRNW, the damaged edge *e* connecting the nodes with the largest weight may be far away from the functional network. The restoration of other damaged edges which constitute the path between the network and the node with the largest weight is a preparation for restoring *e*. These edges always connect nodes with small weights, which lead to a slow increase in recovery level at the early stage of PRNW. But after the initial steps, PRNW has a higher recovery level than that of PR because for each edge restoration in PR, the weight of the repaired periphery is large but not necessarily the largest.

In summary, PRNW has very similar recovery efficiency with GR, and PR is slightly worse than PRNW. Meanwhile, PRNW and PR have lower computational complexity than GR, and are much easier to be applied in simulations. PRNW and PR also have other important merits in policy implication, which will be further discussed in the Discussion section. We also tested different recovery methods when the network is attacked by MA and RA. The results of the numerical simulation are shown in Fig. S3. One can see that the curves of PRNW and PR after MA and RA are totally different from that after LA.

Figure 2(C) displays the recovery level of PRNW after LA, MA and RA. PRNW has the largest recovery efficiency after RA and the smallest recovery efficiency after LA, which implies that it is most difficult to recover the network after LA. In the context of LA, there are isolated nodes in the attack center such that much more edges have to be repaired to connect them to the functional network. While for MA and RA, most isolated nodes are close to the functional network and restoring the connectivity between them and the network requires much less repairs than the case of LA.

Figure 2(D) shows the difference of recovery level between PRNW and RR under three types of attacks. As we can see, the largest difference is found in the case of LA compared to those of MA and RA. In other words, for most values of the edge recovery percentage, PRNW has larger recovery level than RR. Moreover, as we can see in Fig. 2(B), the recovery efficiency of PRNW is much larger than that of RR. It implies that in comparison with MA and RA, strategic recovery methods are much more effective than random recovery after LA.

After revealing the general behaviours of the various scenarios of attacks and repairing, we go on to examine the influence of initial edge damage percentage, network size and nodal weight distribution on the performance of different recovery approaches.

#### Initial Edge Damage Percentage

**Figure 3:**
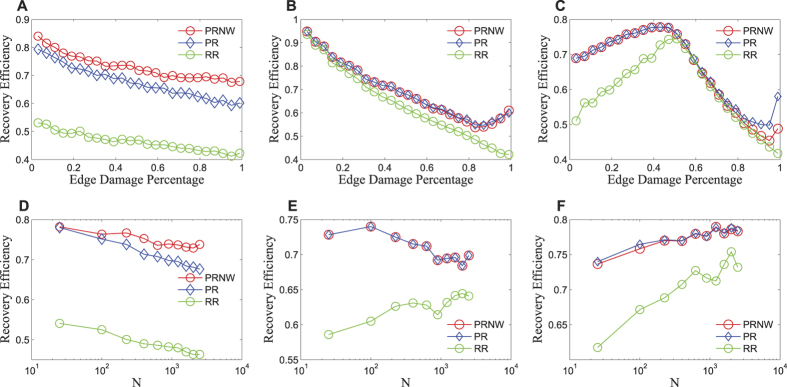
Recovery efficiency of PRNW, PR, RR. (**A**–**C**) respectively under LA, MA, RA with a network size *N* = 2500. (**D**–**F**) respectively under LA, MA, RA with a percentage of damaged edge of 0.45.

As shown in Fig. 3(A), the recovery efficiency of all the three recovery approaches decreases with increasing percentage of initially damaged edges. It implies that network recovery becomes more difficult when more edges are damaged initially. To regain the necessary connectivity, the percentage of recovery edges needs to be larger. PRNW shows a much better performance than PR when the percentage of initially damaged edges is higher, since PRNW is easier to reach the populated nodes than PR. However, the difference between PRNW and RR is relatively stable.

Similar behaviours are found for MA, as shown in Fig. 3(B), except an increasing tail is observed for PRNW and PR in the regime with large initial damage percentage. It is because MA deliberately reduces the whole network functionality, such that the largest sub-networks are always broken down into smaller sub-networks when the percentage of initially damaged nodes is large. Isolated nodes do not emerge until all sub-networks are left with only one edge. That is to say, one node connects one and only one edge. There is a threshold of edge damage percentage (*N* − 1)/2*E* ≈ 0.75, below which the connectivity is likely to have been destroyed but no isolated node exists. The edges connecting the nodes with the largest weight will be restored in priority. In other words, all the surrounding edges of the nodes with the largest weight will be repaired first, followed by those of the second largest weight. Therefore, a large portion of damaged edges will be repaired before the network regains its connectivity, which makes the recovery efficiency of PRNW and PR worse than that of GR in the context of MA, as shown in Fig. S3. When the edge damage percentage is larger than 0.75, the isolated node emerges. With an increasing edge damage percentage, on one hand, the increasing isolated nodes will make it more difficult to repair the network; on the other hand, the hubs are completely isolated in this case when the initial edge damage percentage is larger than 0.75, then connecting one link already regains a lot of network recovery. This happens when damage percentage increases, and more and more isolated hub nodes emerge, so the repairing becomes easier, leading to an increase in network efficiency. Therefore, due to the above contrary effects, with the edge damage percentage increases from 0.75, the recovery efficiency still declines first and then increases.

Compared with MA and LA, the recovery approaches show different characteristics under RA. The recovery efficiencies of PRNW, PR and RR increase first, and then decline when the initial edge damage percentage is 0.5. The explanation of this phenomenon is that under RA, the damaged edges are uniformly distributed throughout the whole network. When the percentage of damaged edge is smaller than 0.5, more important edges are removed when damage percentage increases, but the network is still connected and shows strong robustness. So it will be easier for the network to recover, the more important edges are removed and can be repaired now. In other words, the gain from repairing these important links is higher. As a result, the network efficiency increases. At the same time, the strategic recovery approaches have higher effectiveness than random recovery. On the other hand, when the percentage of the damaged edge is larger than 0.5, the network is separated into several sub-networks and becomes disconnected. In the early stage of repairing, much more damaged edges should be repaired to regain the basic functionality of the network. With an increase in the percentage of initially damaged edges, network recovery becomes more difficult.

When the percentage of initially damaged edges becomes very large, the recovery efficiencies of PRNW and PR also bound back upwards, and their difference with RR becomes larger. The reason is similar to that observed in the case of MA, but with two different characteristics. First, the percentage of damaged edges at which the recovery levels of PRNW and PR start increase under RA is larger than that of MA. This is because there is a higher probability of the existence of several sub-networks which contain more than one edge in RA. This phenomenon leads to a larger number of isolated nodes in RA than that in MA given the same percentage of remaining edges. As a result, more damaged edges have to be repaired to regain the connectivity. Second, the performance of PRNW is worse than that of PR under RA when the percentage of damaged edges is larger than 0.6. The uniform distribution of remaining intact edges makes it much easier for PR to find the nodes with the largest weight.

When the percentage of damaged edges is extremely large (very close to 1), large-scale edge disruption emerges in the whole network, just like a huge localised attack. In this case, a very limited number of edges remain, and the recovery procedures of PRNW and PR after MA and RA are close to those after LA, as shown in Fig. S4. As we can see, the recovery efficiency of PRNW and PR is very sensitive to the percentage of damaged edges, especially for PRNW after RA. The results are significantly different when the percentages of damaged edges are respectively 0.99 and 0.999 under MA and RA.

### Network Size

We then examine the relation between the recovery efficiency and the network size. We show in Fig. 3(D–F) the recovery efficiency of the various recovery approaches after LA, MA and RA with 45% of the edges damaged, as a function of system size *N*. The behaviour of RR is not universal after the various kinds of attack. With the increasing network size, the recovery efficiency of RR declines under LA, but increases under MA and RA. For LA, the recovery efficiency of PR decreases with the increasing network size. In other words, it becomes more difficult for PR to recover a larger network. The same happens for PRNW under LA, as well as for PRNW and PR under MA when network size is small. Under RA, the recovery efficiency of PRNW and PR increases with *N* when *N* is small. It implies that it is easier for PRNW and PR to recover larger networks after RA. Among the three repair approaches, PRNW shows the most stable recovery efficiency with the system size *N*, implying that the performance of PRNW is less affected by scale and can be used in the repairing of large networks. In addition, PRNW significantly outperforms the other two approaches in restoring the networks disrupted by LA. Moreover, deliberate recovery approaches are better than random recovery under MA and RA as the network size increases. Figures S5–S7 further illustrates the recovery efficiency of the various recovery approaches in the parameter space of edge damage percentage and network size after the three kinds of attacks. Analyses are given in SI.

### Nodal Weight Distribution

Based on the recovery procedures of PRNW and PR, it is reasonable to anticipate that population (nodal weight) distribution is one of the key factors that influences the effectiveness of the various deliberate recovery approaches. To further study the influence of nodal weight distribution, we analyse the two-dimensional lattice networks with more homogeneously nodal weight distribution (we have chosen Poisson distribution) for comparison, as shown in Fig. S8. For PRNW and PR, the recovery level increases linearly. There is no abrupt change of recovery level, since the number of nodes with various magnitude of weight is relatively even. In this case, PRNW is not obviously superior to PR. On the other hand, RR is similar to that in the lattice network with heterogeneous weight distribution.

### Real Networks

The recovery of network infrastructure systems from localised attacks induced by natural disasters has drawn wide attention throughout the world. United Nations International Strategy for Disaster Reduction (UNISDR) put forward Sendai Framework for Disaster Risk Reduction on 18 March 2015, which listed damage reduction and resilience development of critical infrastructures as one of the global targets, and regarded high cost-benefit investment to relieve disaster risk as a priority for action. Transportation networks are one of the most typical infrastructures. We thus take Hainan province in China as our empirical study to test the various attacks and recovery approaches, which is separated by the Qiongzhou strait from the mainland of China, making its road network relatively independent. The degree of nodes in the Hainan highway network follows a Gaussian distribution with an average value of 3.14, as shown in Fig. S9, which means the degree is homogeneous and the network is close to a 2-dimensional lattice network. The population near the node is treated as the weight of the node. We choose two disaster scenarios as localised attacks to analyze the resilience of Hainan highway network. The highway network map used in this paper was digitalised from the publication^31^ using ArcGIS 9.3 for deriving its topological information. Demographic data are provided by the Hainan statistical yearbook 2014.

### Earthquake

**Figure 4:**
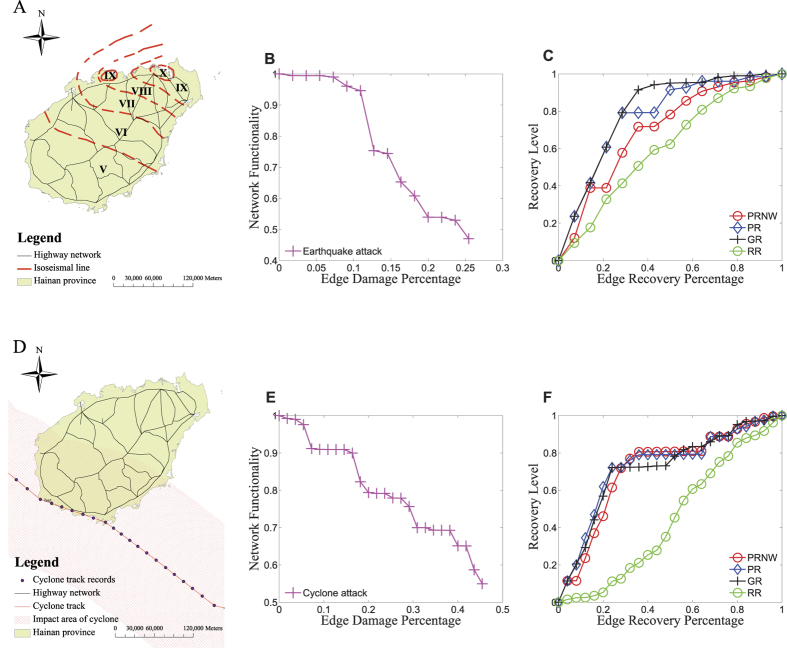
Recovery procedures in a real-world disaster scenario. (**A**) The seismic intensity distribution of the 1605 Qiongshan earthquake in Hainan province of China. (**B**) The degradation process of network functionality under the earthquake. (**C**) The recovery level of the four recovery approaches after the earthquake. (**D**) The affected areas of the second cyclone in 2010 in Hainan province. (**E**) The degradation process of network functionality under the cyclone. (**F**) The recovery level of the four recovery approaches after the cyclone hit. The maps in (**A**,**D**) are drawn using ArcGIS 9.3 (http://www.esri.com/software/arcgis/arcgis-for-desktop).

There was an extraordinary earthquake happened in Hainan in 1605, namely the Qiongshan earthquake, which reached up to a magnitude *X*. It is no doubt that there will be a big catastrophic disaster if earthquake of the same magnitude happens again. Figure 4(A) illustrates seismic intensity distribution of the 1605 Qiongshan earthquake in Hainan province, which is redrawn from the map opened on the website of Hainan seismological Bureau (http://dzj.hainan.gov.cn/zqzq/). The most severely affected area was located in the northeastern part. In Fig. 4(B), the network functionality degradation under earthquake was close to a linear degradation, which is similar to the result of numerical simulations. The recovery procedures of the four recovery approaches under the earthquake are shown in Fig. 4(C). GR has the highest recovery efficiency, followed by PR, then by PRNW. As we can see, RR has the worst effect. PR is better than PRNW, which is different from the numerical simulations. Even though the population in Hainan province is close to a power-law distribution as shown in Fig. S10(a), the vertexes with larger population are located in the coastal areas which are the margins of the network as shown in Fig. S10(b). Therefore, in PRNW, edges connected to nodes with smaller population need to be repaired before the edges connected to nodes with larger population are repaired, which makes PRNW worse than PR.

### Typhoon

Hainan province frequently suffers from typhoons, among which more than 25% of the typhoons are strong tropical cyclones and often disrupt the road network. Highways are sometimes closed in advance of tropical cyclones with wind speed larger than 50 knots in many areas due to strong winds and a mass of wind-borne debris^32^. The hazard can induce physical damage to traffic poles, overhead and roadside signs. We randomly choose one typhoon, the second typhoon in 2010, as a cyclone scenario. The cyclone track is abstracted from China Meteorological Administrations tropical cyclone data center for the western North Pacific basin (http://tcdata.typhoon.gov.cn/), which consists of the location, the maximum sustained wind (MSW), and the minimum sea level pressure at 6 hour intervals. The track is interpolated into one hour intervals by Inverse Distance Weighting method. Its maximum sustained wind was 35 *m*/*s*. It was across the west-southern part of Hainan Island. This tropical cyclone disrupted about 40% of highway road segments. The detailed calculation of the affected area of the cyclone is shown in SI. We also examined different cyclones and the results are similar.

Figure 4(D) displays the affected areas of the cyclone in the Hainan province. The affected areas are located in the southwestern part. Network functionality degradation under the cyclone is also close to a linear degradation, as shown in Fig. 4(E). In Fig. 4(F), the recovery procedures of four recovery approaches are shown. The deliberate repair approaches PRNW, PR and GR show a similar profile in the recovery level, which is much better than that of RR. These results are similar to the results obtained from numerical simulations.

## Discussion

This paper provides insight into deliberate and strategic recovery approaches on geographical networks under localised attacks. Literatures^23^, as well as Fig. 2(A), imply that localised attacks indeed do not ruin the system as much as malicious attacks. Nevertheless, we need a good strategy to recover the system from localised attacks, and our results show that it is difficult to recover the system after localised attacks compared with malicious attacks and random attacks. In comparison with strategic recovery, the effect of random recovery is less than satisfactory. Strategic recovery approaches such as the preferential recovery based on nodal weight (PRNW) and periphery recovery (PR) are shown to be much more effective after localised attacks, and the results are well supported by numerical simulations and simulations on real-world disaster scenarios.

The philosophy behind PRNW is consistent with the demand of real-world disaster risk reduction. After the occurrence of natural disasters, the first 72 hours are sometimes quoted as the golden time and is the most valuable time for rescue. There is an urgent need to strategically recover the infrastructure network in a timely and efficient manner. The proposed PRNW shows a low computational complexity, and a high efficiency in connecting the most populated region in a short time. On one hand, PRNW is concerned with the holistic connectivity to ensure every node is connected and accessible to the functional component of the network. On the other hand, in the premise of connectivity, the disrupted edges linking with the largest weighted nodes are preferentially repaired, which enhances the recovery level in the early stage. While GR always repairs edges to maximise the recovery in the network functionality, there may still exist isolated nodes a long time after the incidence. Most importantly, PRNW is very similar to GR in terms of recovery effectiveness under LA for the whole recovery process. In addition, in the early stage, the recovery level of PRNW is higher than that of GR, which implies that when the recovery resource is too limited to ensure all the isolated nodes to connect the network, PRNW can still provide a reasonable solution with high cost-benefit ratio.

Most natural hazards, not merely earthquakes and cyclones, are typical localised attacks. Our findings provide a scientific understanding to recognise the intrinsic recovery mechanism from localised attacks as well as different characteristics of malicious attacks and random attacks. These findings also offer important support for risk management for a wide range of real world systems. The proposed PRNW provides solid scientific evidence for strategic resource allocation to optimise the recovery process. It is an intelligent post-disaster recovery method, which can greatly shorten the time needed to restore system function and reduce the associated losses, especially for infrastructure systems. Similar applications can be extended to ecological systems and social systems if their function mechanism of can be modelled precisely.

Future studies can be extended to investigate the impact of geographical distribution of population on the consequences of localised attacks and on the efficiency of recovery strategies. We only consider topological metrics on one independent infrastructure network in this work. Infrastructure networks such as water supply networks, electric power grids, transportation networks involve specific processes of flow dynamics. There also exist various interdependencies among coupling critical infrastructure networks^33^,^34^, including physical interdependency, cyber interdependency, geographic interdependency and logical interdependency^35^. The combination of dynamics and interdependencies can propagate and amplify a small amount of failures, inducing a series of cascades effects^36^,^37^,^38^,^39^, and are studied as cascading failures in interdependent networks^10^. In further investigation, post-disaster recovery of infrastructure network should also take systemic dynamics and interdependencies into account. Besides, some realistic infrastructure networks show different characteristics of cascading failures. Pahwa *et al.*^40^ pointed out that due to long-range nature of electric interaction, a first order phenomenon can be predicted using a simple mean-field model from line overloads angle, and an increase in system size leads to more abrupt breakdown. Yet, further investigations are needed for the recovery dynamics of large power grids after a black-out^41^,^42^.

## Methods

### Nodal Weight Distribution

We adopt two probability distributions to respectively describe the homogeneous and heterogeneous nodal weight, i.e. population.

(a) Power-law distribution:


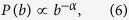


where *b* is the nodal weight and *α* is the exponent determining the heterogeneity. In the numerical simulations, we set *b* to be between 1 and 1000 and *α* = 1.

(b) Poisson distribution:


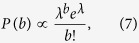


where *b* is the nodal weight and *λ* is the mean value of *b* in the distribution *P*(*b*). In this paper, we set *λ* = 5.

### Network Recovery Approach

Four different approaches are adopted on network recovery.

(a) Preferential Recovery based on Nodal Weight (PRNW):


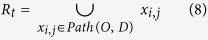


where *x*_*i*,*j*_ denotes the edges between node *i* and *j*, *Path*(*O*, *D*) is the set of edges on the shortest path between nodes *O* and *D*, such that *D* is the node with the maximum weight among all isolated nodes, and *O* is a node connected to the functioning component of the network and is closest to *D*. *R*_*t*_ is thus a set of edges which are repaired at *t*^*th*^ iteration. And *x*_*O*,*i*_ and *x*_*j*,*D*_ are respectively the first and the last edges on the shortest path from *O* to *D*. The computational complexity of PRNW is *O*(*N*^3^).

(b) Periphery Recovery (PR):


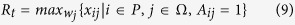


where *x*_*i*,*j*_ denotes the edges between node *i* and *j*, *w*_*j*_ is the weight on node *j*, *P* is set of nodes on periphery or nodes that are connected to the functioning component of the network and has at least one damaged edge, and Ω is the set of nodes that are isolated. *R*_*t*_ is the set of edges which are repaired at the *t*^*th*^ iteration. The computational complexity of PR is *O*(*N*^2^).

(c) Greedy Recovery (GR):


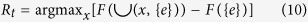


where {*e*} is the set of functioning edges at *t* − 1^*th*^ iteration, and *F*({*e*}) is the network functionality with {*e*}, *R*_*t*_ is the set of edge which are repaired at *t*^*th*^ iteration. The edge *x* which maximises the increase in network functionality is restored at each iteration. The computational complexity of GR is *O*(*N*^4^).

(d) Random Recovery (RR): This is the simplest method where a damaged edge is randomly chosen to be restored in each time step. The computational complexity of RR is *O*(*N*).

## Additional Information

**How to cite this article**: Hu, F. *et al.* Recovery of infrastructure networks after localized attacks. *Sci. Rep.* **6**, 24522; doi: 10.1038/srep24522 (2016).

## Supplementary Material

Supplementary Information
