# Supplementary material for: Recovery of infrastructure networks after localised attacks
Source: Sci Rep. 2016 Apr 14;6:24522. doi: 10.1038/srep24522 (PMC4830952; doi:10.1038/srep24522)
Supplement: Supplementary Information [file srep24522-s1.pdf]

Supplementary Information of " Recovery of infrastructure networks after localised attack"

Fuyu Hu, Chi Ho Yeung, Saini Yang, Weiping Wang and An Zeng

I. SUPPORTING FIGURES

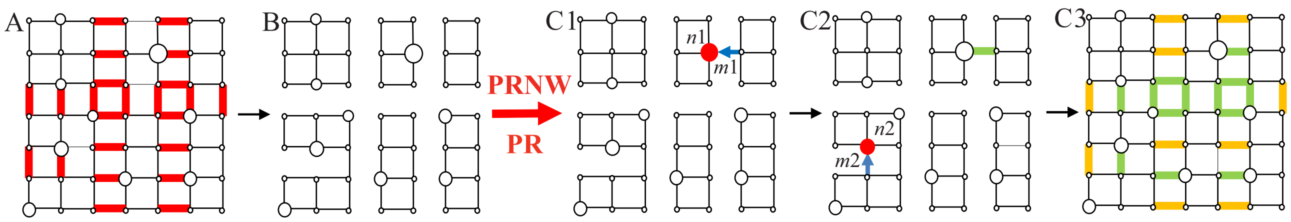

Fig. S 1: (Colour online) Deliberate recovery process of heterogeneously weighted lattice network under malicious attack. (A) illustrates the damaged edges dyed with red which have larger betweenness concentrate in the center of the network. (B) is the remaining network after attack and it has been separated into several parts. (C1) Node $n1$ has largest weight and is adjacent to a damaged edge $m1$. $m1$ will be repaired first. (C2) the repaired edge is dyed with green and $m2$ will be the next repaired edge. (C3) when all the edges connecting to the nodes with larger weight are repaired, the remaining edges will be repaired randomly.

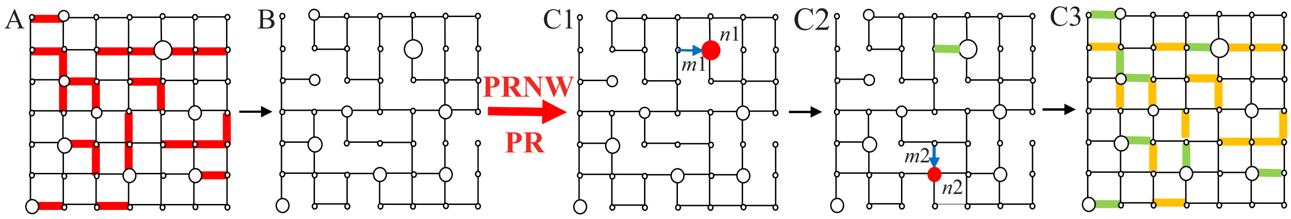

Fig. S 2: (Colour online) Deliberate recovery process of weighted lattice network under random attack. (A) illustrates the damaged edges coloured in red are randomly distributed in the network. (B) is the remaining network after attack. We can find that nearly no isolation nodes after random attack. (C1) The edges adjacent to the nodes with large weight will be repaired in priority. $n1$ has the largest weight, so edge $m1$ will be restored first. (C2) The repaired edge is coloured in green. Then node $n2$ has the largest weight and it connects edge $m2$. (C3) After repairing the edges connecting larger weighted nodes, we randomly repaired the remaining edges.

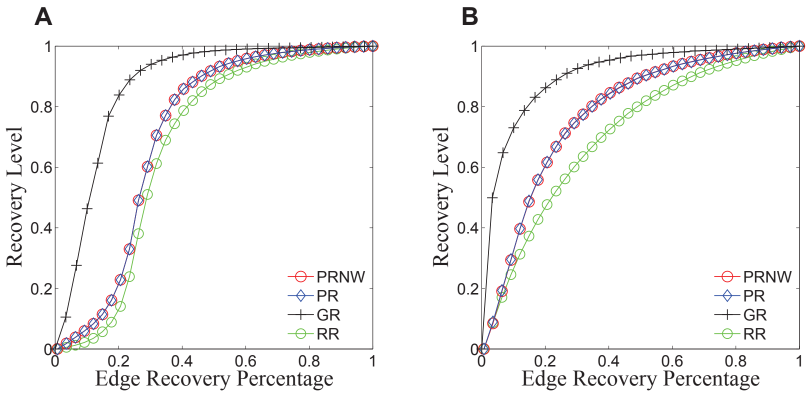

Fig. S 3: (Colour online)Recovery process of PRNW, PR, GR, RR (A) under MA and (B) under RA.

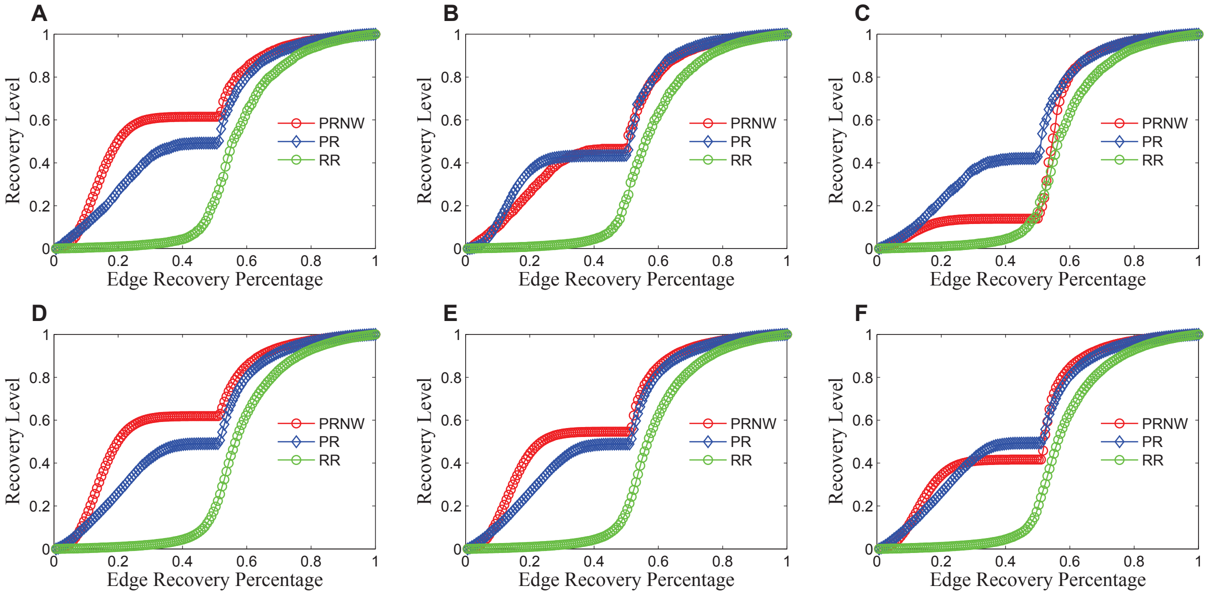

Fig. S 4: (Colour online)Recovery level of PRNW, PR and RR. (A-C) respectively under LA, MA and RA, edge damage percentage=0.99. (D-F) respectively under LA, MA and RA, with edge damage percentage =0.999.

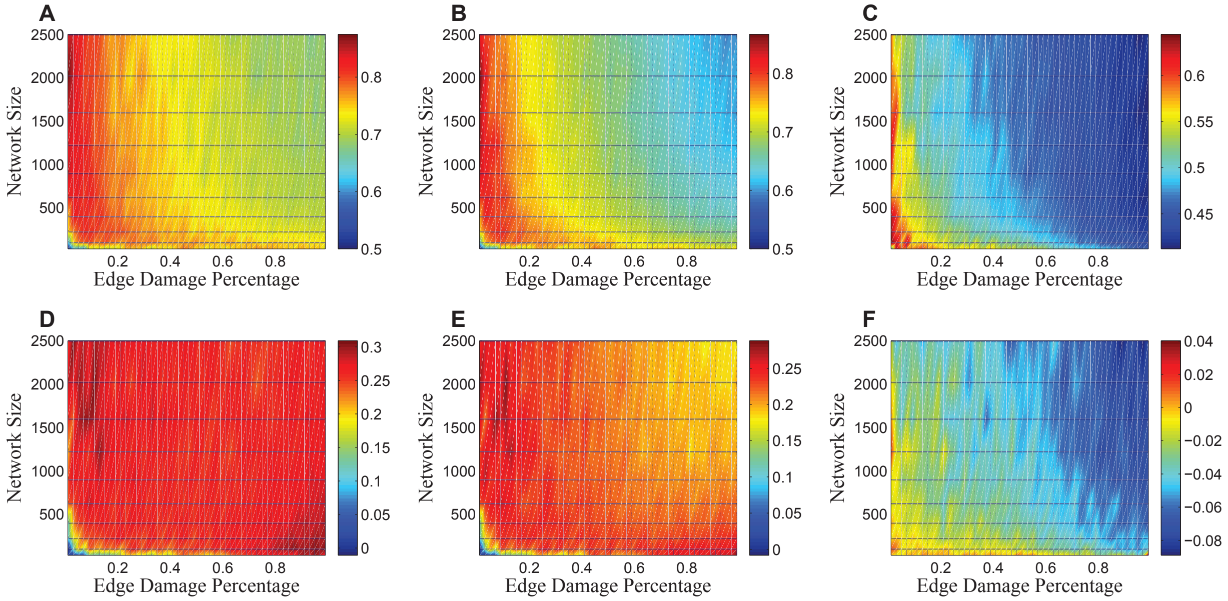

Fig. S 5: (Colour online) Recovery efficiency of the network with heterogeneously nodal weight under localised attack in the parameter space of edge damage percentage and network size. (A) RE_{PRNW}. (B) RE_{PR}. (C) RE_{RR}. (D) $RE_{PRNW} - RE_{RR}$. (E) $RE_{PR} - RE_{RR}$. (F) $RE_{PR} - RE_{PRNW}$.

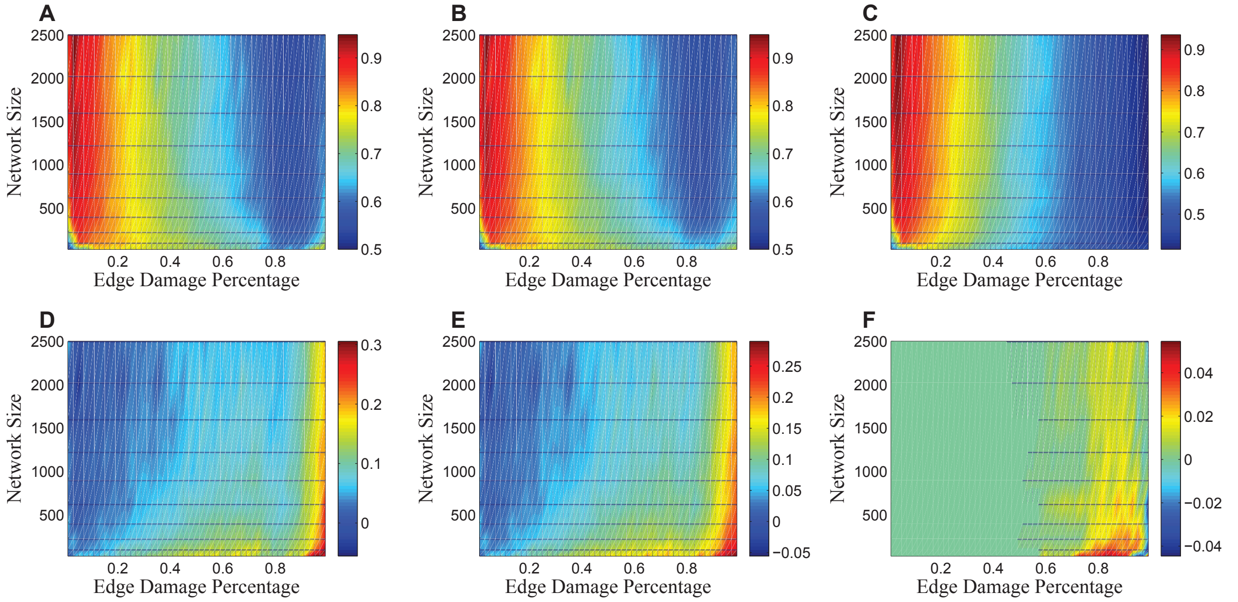

Fig. S 6: (Colour online) Recovery efficiency of the network with heterogeneously nodal weight under malicious attack in the parameter space of edge damage percentage and network size. (A) RE_{PRNW}. (B) RE_{PR}. (C) RE_{RR}. (D) $RE_{PRNW} - RE_{RR}$. (E) $RE_{PR} - RE_{RR}$. (F) $RE_{PR} - RE_{PRNW}$.

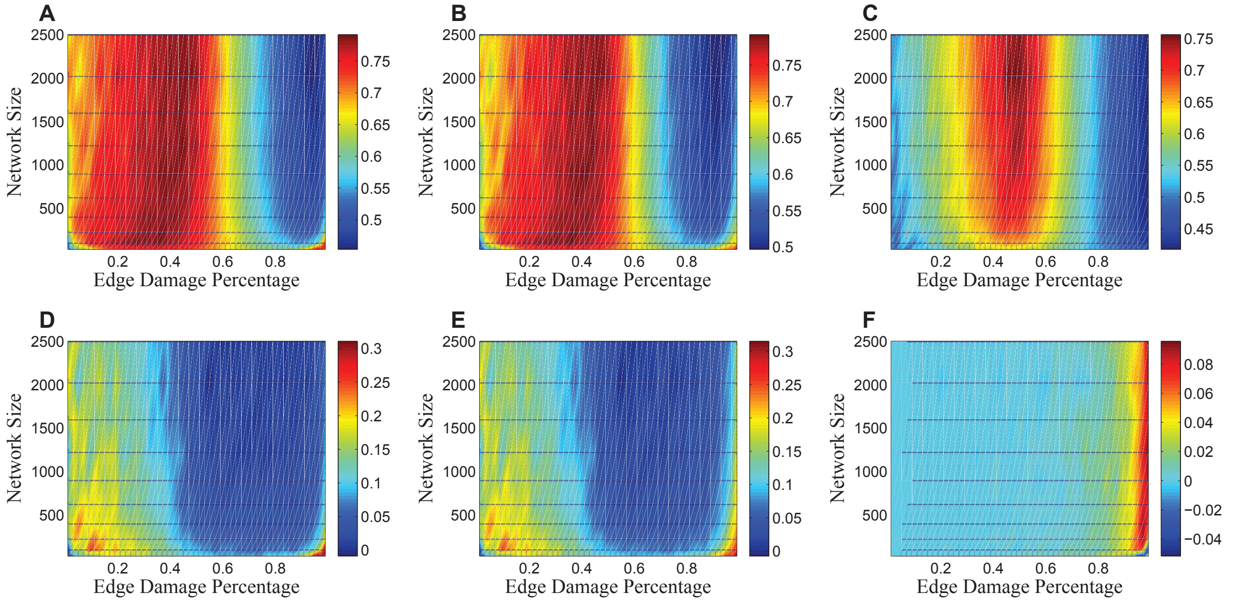

Fig. S 7: (Colour online) Recovery efficiency of the network with heterogeneously nodal weight under random attack in the parameter space of edge damage percentage and network size. (A) RE_{PRNW}. (B) RE_{PR}. (C) RE_{RR}. (D) $RE_{PRNW} - RE_{RR}$. (E) $RE_{PR} - RE_{RR}$. (F) $RE_{PR} - RE_{PRNW}$.

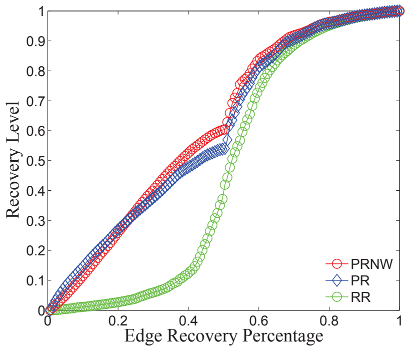

Fig. S 8: (Colour online) Recovery process of the homogeneously weighted network (edge damage percentage = 0.4)

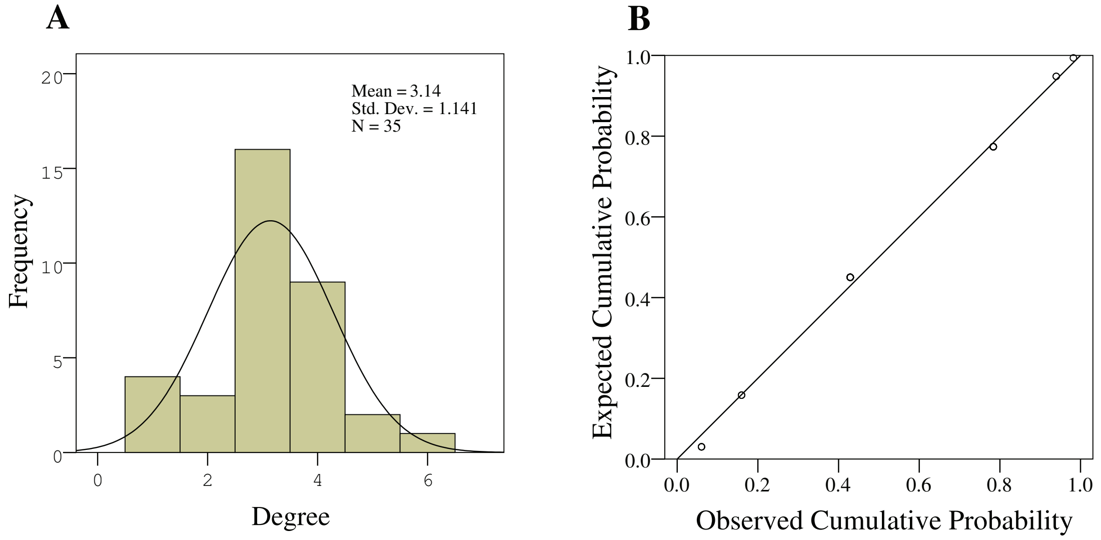

Fig. S 9: (Colour online) (A) The degree distribution of highway network in Hainan province, China. (B) Normal P-P Plot of node degree of highway network in Hainan province. The relation between observed cumulative probability and expected cumulative probability is close to linear, which can validate that the degree distribution of highway network follows a Gaussian distribution.

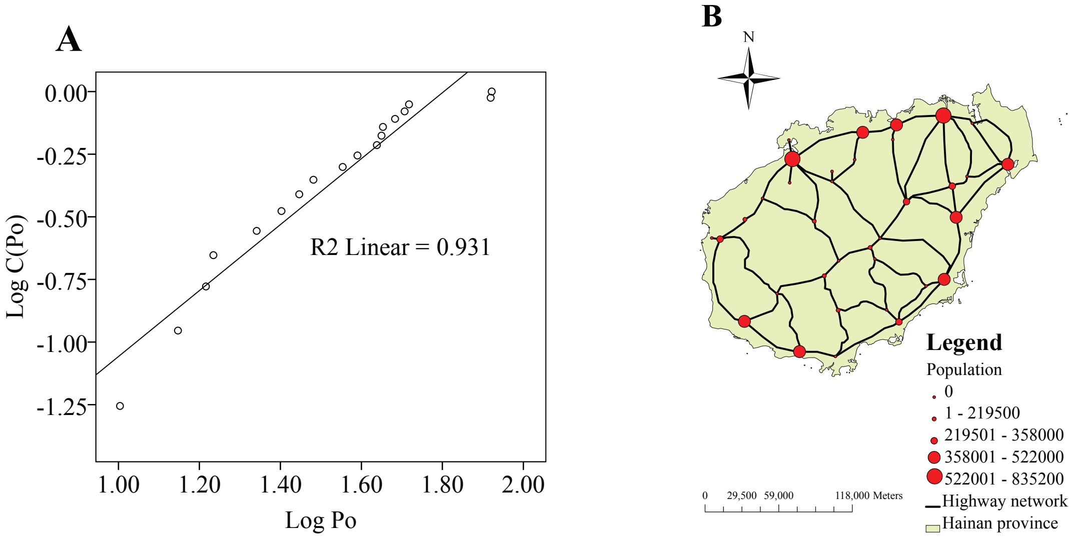

Fig. S 10: (Colour online) (A) Accumulated probability C distribution of population in Hainan province. It displays that population follows power-law distribution. Po denotes population. (B) The spatial population distribution of Hainan province, China. The map was generated using ArcGIS 9.3 (http://www.esri.com/software/arcgis/arcgis-for-desktop). Except a few vertexes with large population, most vertexes have small population.

II. SI TABLES

TABLE. S I: The relationship between seismic intensity and peak ground acceleration

PGA(g)	< 0.05	0.05	0.1	0.15	0.2	0.3	≥ 0.4
SI	$<$ VI	VI	VII	VII	VIII	VIII	$\geq$ IX

III. RECOVERY PROCESS OF PRNW, PR, GR, RR UNDER MA AND RA

As shown in Fig. S3, because damaged edges induced by MA and RA both are distributed throughout the whole network, rather concentrated into a local domain. No isolated nodes are generated in the process of MA and RA, so there only exists two stages in the recovery process for PRNW and PR in the context of MA and RA. Under MA, the first stages of PRNW and PR are to repair the edges connecting larger weighted nodes, through which the network becomes connective again, and simultaneously the distance between larger weighted nodes and others can be shortened. The second stage is similar to the fourth stages of PRNW and PR under LA. Under RA, the two stages of PRNW and PR are similar to the third and fourth stages of PRNW and PR in the context of LA. Apart from the different positions of inflection points, the curve styles of recovery level of different recovery approaches under both MA and RA are very similar. Though GR has an obvious advantage over PRNW and PR, we can still use the latter approaches as effective and timely recovery methods to qualitatively exploit the mechanism of recovery behaviour under different attacks.

IV. PATTERN ANALYSIS FOR RECOVERY EFFICIENCY OF THE HETEROGENEOUSLY WEIGHTED NETWORK IN THE PARAMETER SPACE OF EDGE DAMAGE RATIO AND NETWORK SIZE

As shown in Fig. S5, under LA, the recovery efficiency of PRNW decreases as the network size or edge damage percentage increase. With ascending edge damage percentage, the degradation speed of recovery efficiency of PRNW in a large network is greater than that in a small network. With increasing network size, the degradation speed of recovery efficiency of PRNW in a network with large edge damage percentage is greater than that with small edge damage percentage. PR and RR also have the similar characteristic and PR is the most obvious one. The distribution of $RE_{PRNW} - RE_{RR}$ is even in parameter space of edge damage percentage and network size. $RE_{PR} - RE_{RR}$ becomes slightly different with increasing edge damage percentage, so does $RE_{PR} - RE_{PRNW}$. PRNW and PR are much better than RR. Under LA, PR is slightly better than PRNW if the network size and edge damage percentage are small enough. In most cases, PRNW is slightly better than PR.

In Fig. S6, under MA, when the edge damage percentage is very large, $RE_{PRNW} - RE_{RR}$ (D), $RE_{PR} - RE_{RR}$ (E), $RE_{PR} - RE_{PRNW}$ (F) turn to be large. In the space of very small edge damage percentage and very large network size, RR is better than PRNW and PR. In the remaining situations, strategic recovery is slightly better than random recovery. For PRNW and PR, if network size is small, the variation of recovery efficiency is very small with increasing edge damage percentage; otherwise, the value is quite large. But for RR, the variation is independent of network size.

In Fig. S7, under RA, when edge damage percentage is large, $RE_{PR} - RE_{PRNW}$ (F) becomes larger; when edge damage percentage is small, $RE_{PRNW} - RE_{RR}$ (D) and $RE_{PR} - RE_{RR}$ (E) are large; otherwise, the differentials are small. For any network size, with increasing edge damage percentage, the variation of recovery efficiency of PRNW, PR and RR are very similar. At around edge damage percentage 0.5, the recovery efficiency reaches the peak value and gradually declines in two sides. For RR, the degradation speeds of two sides are similar. For PRNW and PR, when edge damage percentage is smaller than 0.5, the degradation speed is smaller than that when edge damage larger than 0.5.

V. THE CALCULATION OF IMPACT AREA OF TYPHOON SCENARIO

The radius of 50 knot winds or greater (RW_{50kt}) of tropical cyclone is defined as its impact areas. Miller model [1] is used to calculate RW_{50kt}:

$$RW_{50kt} = RMW \frac{v_m}{v_{50kt}}^{\frac{1}{y}}, \tag{1}$$

Where RMW denotes the radius of the maximum wind speed; V_m is the maximum wind speed; V_{50kt} denotes the wind speed which equals to 50 knots; y is an empirical constant which equals to 0.35 in this case.

To determine the RMW, we refer to the adaptable regression model proposed by Lin and Fang [2] which quantified the relationship between the radius of maximum wind speed and central pressure deficit by using the best track data set (2001 to 2009) from the Joint Typhoon Warning Center in the United States:

$$RMW = -18.04 In\phi + 110.22, \tag{2}$$

Where ϕ denotes the central pressure deficit, namely the difference between sea level pressure and minimum sea level pressure.

We simulated the procedure of tropical cyclone landfalling in Hainan province by ArcGIS Engine second development, as shown in Figure 3.

VI. THE SEISMIC INTENSITY IN EARTHQUAKE SCENARIO

Table S1 shows the relationship between seismic intensity (SI) and peak ground acceleration (PGA). Jia [3] concluded that the highway is very likely to encounter large seismic disruption when PGA surpasses than $0.3g$; it will be suffered from moderate damage when PGA is the value between 0.2 and $0.3g$. It will bear minor damage when PGA is smaller than $0.2g$. Therefore, we treat all the road segments as failure when it encounters PGA larger than $0.3(SI >= VIII)$, no failure when road segments encounters PGA smaller than $0.2(SI <= VII)$, and failure with a probability when road segments encounters PGA between 0.2 and $0.3g$.

[1] Miller,B.I. Characteristics of hurricanes. *Science* **157**, 1389-1399(1967).
[2] Lin,W. & Fang, W.H. Regional Characteristics of Holland B Parameter in Typhoon Wind Field Model for Northwest Pacific. *Tropical Geography(in Chinese)* **33(2)**, 124-132(2013).
[3] Jia, X.L. Syntax of referencing in *Study on Theory and Method of Highway Alignment Selection in High Seismic Intensity Canyon Region(Doctoral Dissertation).* 62 (Chang'an University, Xi'an, China, 2013).
